# Color preference of *Sergentomyia minuta* (Diptera: Phlebotominae) determined using *Flebocollect* Do It Yourself light traps based on LED technology

**DOI:** 10.1007/s00436-022-07720-3

**Published:** 2022-11-21

**Authors:** Marcos López de Felipe, Eva Pérez, Rosa Gálvez

**Affiliations:** 1 Flebocollect Medical Entomology Citizen Science Group, Madrid, Spain; 2 Department of Specific Didactics, School of Education and Teacher Training, Universidad Autónoma de Madrid, Madrid, Spain

**Keywords:** *Phlebotomus*, *Sergentomyia*, CDC, Flebocollect, Light preference

## Abstract

Whether phlebotomine sand flies show a preference for different light colors remains controversial. As light-capture methods are widely used to study sand flies, knowing the visual stimuli they respond to could help the design of novel control tools to prevent their attraction to hosts. We have detected a significant preference of male *Sergentomyia minuta* for green and red light sources. Accordingly, male *S. minuta* were 2.16 and 2.01 times more likely to be lured by Flebocollect model traps with green and red diode-lights, respectively, than the commercial CDC traps. Flebocollect traps are homemade light traps developed through citizen science. Dipterans are widely considered unable to distinguish the color red so this finding was unexpected. To our knowledge, this is the first description of a color preference in a species of the genus *Sergentomyia*. Our research also confirms the great potential of Flebocollect light traps for use in medical entomology studies.

## Introduction

Phlebotomine sand flies (Diptera: Psychodidae) are small insects that can transmit diseases caused by different pathogens to a great variety of vertebrates during blood feeding. For reasons of public and veterinary health, protozoans of the genus *Leishmania* are the most relevant of these pathogens (Killick-Kendrick 1999). In the Mediterranean basin, the main vector of visceral leishmaniasis, caused by *Leishmania infantum* Nicole, 1908, is *Phlebotomus perniciosus* Newstead, 1911 (Gálvez et al. 2018). In the study area along with other regions of Spain, *P. perniciosus* is the most abundant species followed by *Sergentomyia minuta* Rondani, 1843 (Gálvez et al. 2010; Prieto et al. 2021). This study is aimed at evaluating the effect of different diode light sources in the effect of capturing phlebotomine sand flies through the employment of Flebocollect Do It Yourself light traps.

### How do insects perceive their surroundings and what attracts them? The basis of visual stimuli in insects

As seen in other crepuscular and nocturnal insects such as mosquitoes (Diptera, Culicidae) or moths, phlebotomine sand flies show positive phototropism, meaning they are attracted to artificial light sources (Alexander 2000). This has been traditionally exploited by medical and veterinary entomologists worldwide through the attraction of dipterans to traps by light lures. The best known examples are the Center for Disease Control and prevention (CDC) light traps (Alexander 2000). These commercial models have an incandescent halogen light source positioned over a fan that sucks flying insects into a contention mesh bag. In addition, chemicals and organic compounds such as dry ice or yeast and sugar (Smallegange et al. 2010) can be used as sources of carbon dioxide to increase the effectiveness of these traps (Alexander 2000). Other kairomones such as octenol or the commercially available BG-Mesh Lure (BGML: lactic acid, caproic acid, and ammonia) have been proven effective for attracting New World phlebotomine sand flies (Andrade et al. 2008).

The compound eye is usually the main adult insects’ visual structure. It is composed of different subunits or ommatidia, each of which contains a rhabdom, the ultrastructure responsible for light perception (Gullan and Cranston 2014). In the case of dipterans, this rhabdom is considered to be open, meaning that two separate visual pathways exist in each ommatidium (Chapman 1998; Lunau 2014; Schnaitmann et al. 2020). The rhabdom contains the insects’ visual pigments or rhodopsins (Chapman 1998). Different types of rhodopsin break at different wavelengths, and this enables color light perception in insects (Schnaitmann et al. 2020). Light perception is thus the result of the rupture of this visual pigment due to modification of its structure by a specific range of photon wavelengths or colored light sources (Mellor et al. 1996).

### How does light intensity affect phlebotomine sand flies? Impacts of light on sand fly ecology

Studies investigating sand fly light and color detection are limited in the bibliography. Phlebotomine sand flies are mainly crepuscular, as they usually feed from sunset to midnight, when the temperature cools and relative humidity starts to increase (Lucientes et al. 2005). As early as in 1971, Chaniotis and colleagues detected increasing numbers of New World phlebotomine sand fly bites with decreasing light intensity (Chaniotis et al. 1971). Moonlight also seems to influence sand fly captures, although studies examining this factor have provided disparate results. Thus, while higher capture rates of various phlebotomine New World species and *Phlebotomus orientalis* Parrot, 1936, were recorded in Brasil and Ethiopia, respectively during new-moon nights than full-moon nights (da Silva et al. 2020; Gebresilassie et al. 2015), in the cases of *P. perniciosus* and *S. minuta* in Sicily (southern Italy), more individuals were captured in full-moon than new-moon nights employing the novel LAIKA 4.0 model light trap (Gaglio et al. 2014).

### What colors do phlebotomine sand flies prefer?

How different organisms perceive their surroundings is an interesting yet complex topic of research. As an initial approach, we can try to understand the effects of different light stimuli on visual perception. In an electroretinogram study performed by Mellor in 1996, *Lutzomyia longipalpis* Lutz & Neiva, 1912 was found to show a main absorption peak for ultraviolet light and a second smaller peak for blue-green-yellow wavelengths (Mellor et al. 1996). Similar observations have been reported for other dipterans such as *Drosophila* spp. (Schnaitmann et al. 2020), *Aedes* spp. (van der Kooi et al. 2021), or *Culicoides* biting midges (González et al. 2016). As such, different explanations for color light preferences have been proposed in the past. The attraction of mosquitoes to short and medium wavelengths has been previously associated with the selection of oviposition sites (van der Kooi et al. 2021). Also, ultraviolet light perception is usually associated with pollinator insects due to their need for plant detection (Schnaitmann et al. 2020). Such is also the need of phlebotomine sand flies, as both male and females feed from sugar sources such as nectar, honeydew, and/or phloem sap accessed by piercing leaves and steams with their mouthparts (Abbasi et al. 2018). Several studies have addressed color preferences both in New and Old World phlebotomine sand flies, although results have been so far inconclusive (Table 1).

**Table 1:** Main characteristics and results of studies addressing light color preferences of New and Old World phlebotomine sand flies

Color preference	Species	New or Old World	Country	Trap model or experimental procedure	References
Green	Several species	New World	Brazil	CDC	(da Silva et al. 2016, 2015)
Green and blue	Several species	New World	Brazil	Not specified	(da Silva et al. 2022)
Ultraviolet (UV)	*Lutzomyia longipalpis*	New World	Brazil	Electroretinogram	(Mellor et al. 1996)
	*Lutzomyia longipalpis*	New World	Brazil	In vitro* (light tunnel)*	(Mellor and Hamilton 2003)
	Unspecified	New World	Colombia	CDC	(Cohnstaedt et al. 2008)
	*Phlebotomus perniciosus*	Old World	Italy	LAIKA 4.0	(Gaglio et al. 2017)
	*Phlebotomus* spp.	Old World	Iraq	CDC	(Burkett et al. 2007)
Incandescent bulb	*Lutzomyia* spp.	New World	Mexico	CDC and Disney	(Rodríguez-Rojas et al. 2016)
Red	*Lutzomyia shannoni*	New World	USA	Mosquito Magnet-X	(Mann et al. 2009)
	*Phlebotomus papatasi*	Old World	Egypt	CDC	(Hoel et al. 2007)
Blue-green–red	*Lutzomyia vexator*	New World	USA	Mosquito Magnet-X	(Mann et al. 2009)
No preference	*Lu. longipalpis*, *Migonemyia migonei*, and *Nyssomyia whitmani*	New World	Argentina	REDILA	(Fernández et al. 2015)

#### Color light preference studies in New World phlebotomine sand flies

In three different studies conducted in Brazil, green LED light provided the higher capture rates (Silva et al. 2015, 2016), and this was attributed to a larger effect area and a reduced detrimental effect of moonlight on green diode light sources than conventional CDC light traps (da Silva et al. 2020). Blue has also been suggested as to capturing the same abundance and diversity of sand flies as green and incandescent light sources, whilst red and ultraviolet seem ineffective in capturing those insects (da Silva et al. 2022). Another Brazilian study revealed in vitro the preference of *Lu. longipalpis* for UV and blue-green-yellow wavelength light (Mellor and Hamilton 2003). In Mexico, Rodríguez-Rojas and colleagues observed a preference of several *Lutzomyia* species for conventional incandescent CDC light traps compared to those same models but modified to contain LED light sources (Rodríguez-Rojas et al. 2016). Using Mosquito Magnet-X traps, Mann and colleagues observed a preference for red and blue-green–red LED light for *Lu. shannoni* Dyar, 1929 and *Lu. vexator* Coquillet, 1907 respectively in Florida, USA, although no statistical evidence was provided (Mann et al. 2009). In a study in which commercial CDC traps and the REDILA trap (homemade light trap developed by the Argentinean Leishmaniasis Research Network) set up to emit white and black light were used to capture *Lu. longipalpis*, *Migonemyia migonei* França, 1920, and *Nyssomyia whitmani* Antunes & Coutinho, 1939 in Argentina, no differences in efficiency were observed (Fernández et al. 2015). Another modification of the CDC light trap of interest is the “lightning LED technology trap” developed by Cohnstaedt and colleagues in Colombia. This modification was based on placing a LED-light platform over the CDC trap. This enabled the authors to set up between 4 and 16 diodes over the trapping fan and to freely select the color, intensity and viewing angle of the light source. Results indicated 31% and 42% more captured phlebotomines using the modified CDC with 4 and 16 UV-LED bulbs respectively, compared to the conventional CDC light trap (Cohnstaedt et al. 2008).

#### Color light preference studies in Old World sand flies

In studies conducted on Old World sand flies, *Phlebotomus papatasi* Scopoli, 1786, in Egypt showed an unexpected strong preference for red LED light (Hoel et al. 2007) whilst *P. perniciosus* in Italy showed a preference for the UV-light emitted by the novel LAIKA 4.0 model light trap (Gaglio et al. 2017). Similar results were observed in another series of studies conducted during US military operations in Iraq, in which a general preference of *Phlebotomus* spp. for UV-light modified CDC traps was detected (Burkett et al. 2007).

## Methodology

### Study site

The research was conducted in the Animal Shelter of Torrelodones (Comunidad de Madrid, Spain) located at 845 mamsl and characterized by an arid meso-Mediterranean climate (Rivas-Martínez 1983), where the highest phlebotomine sand fly densities of the municipality have been detected (Prieto et al. 2021). The animal shelter is surrounded by an urban area and a rural environment, characterized by the presence of the municipality’s cemetery and by a field that grades from a weathered scrub with sporadic pine and cypress trees (*Pinus pinea* and *Cupressus* spp.) over a granite soil to a dense holm oak forest (*Quercus ilex*) over a matrix of sands (Molina and Lamana 2018).

### Study design

**Fig. 1:**
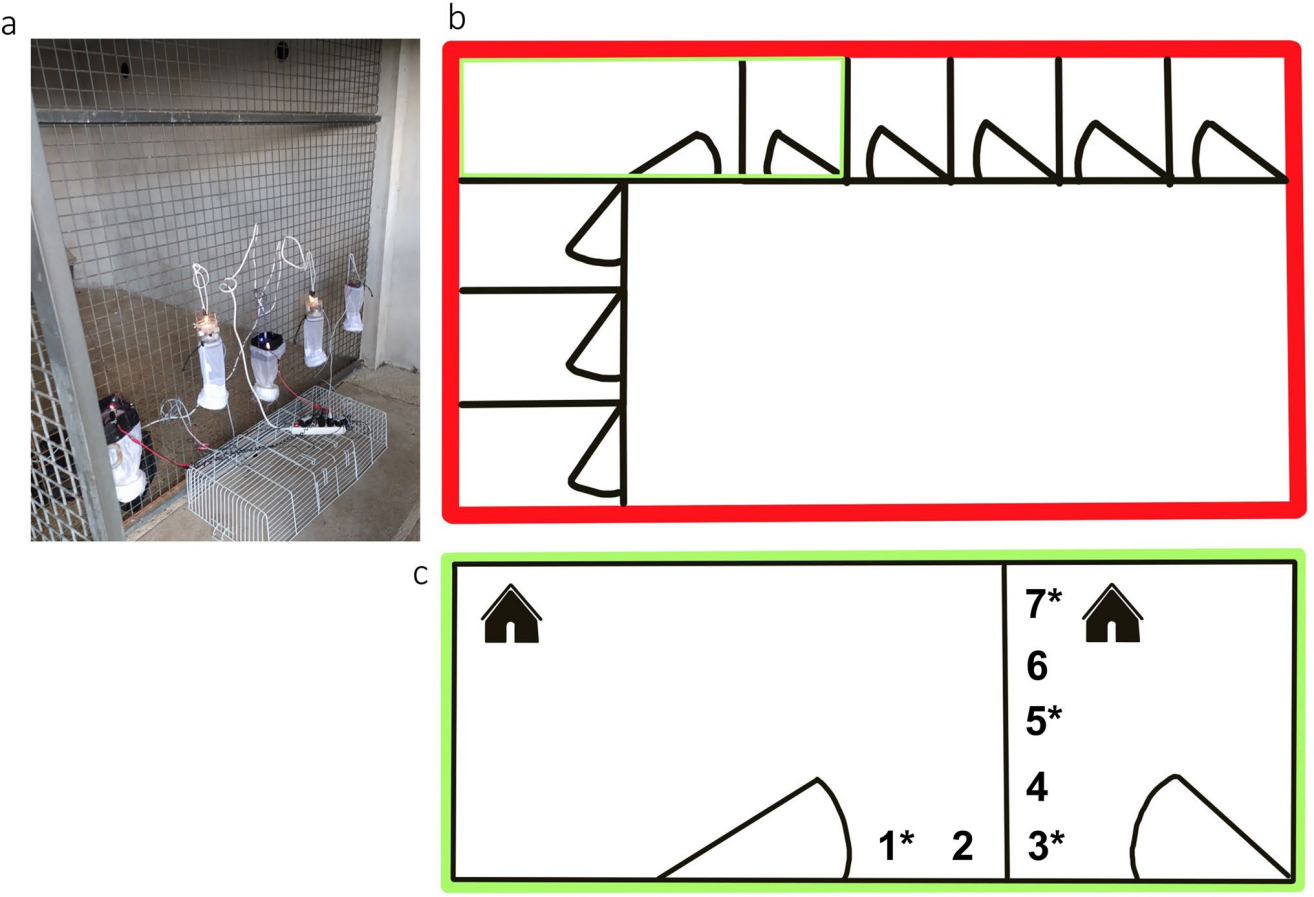
Traps set up to attract sand flies at the Animal Shelter of Torrelodones (Madrid, Spain). **a** Photo of a trapping site; **b** diagram of the shelter; **c** diagram of the two adjacent kennels where the traps (1–7) were installed forming an inverted “L.” *Specific sites where the four Flebocollect test traps were placed

Four Flebocollect model traps (Fc) were constructed (Fc1-Fc4) from recycled materials as described elsewhere (Gálvez et al. 2022) including four types of interchangeable circuits consisting of different color 5 mm, rounded, 2-pin diode diffused lights: blue, green, red, and yellow (luminous intensity 6000–10,000 mcd and wavelengths not specified by manufacturer). Three commercial miniature CDC light traps (BioQuip #2836BQ-6VDC; 6 V, 3.5 A, as recommended by provider) with an incandescent light source were employed as control. The homemade traps employed a 12 V, 3.5 A power supply connected to an electric plug mounted on a wall. All the traps were installed 0.50 m above the ground inside two empty kennels sided by a kennel where a dog was kept, and at 0.75 m above the ground (Fig. 1). As such, traps were placed in the same vision range of phlebotomine sand flies, since the maximum distance of the attraction of the light appears to be 2 m in *P. ariasi* (Killick-Kendrick et al. 1985) and 6 m in *Lutzomyia longipalpis* (Valenta et al. 1995). This way, traps are not completely independent from one another, and a true color preference among traps can be studied.

The traps were set at sunset (20:00 h) and were retrieved the following day after dawn (8:00) over 11 nights between July and September 2020. Both the Flebocollect light traps and diode light circuits were rotated at their corresponding testing sites as specified bellow (Table 2). To assess a possible decline in trapping efficiency over time, Fc1 was set as an Fc control trap, and capture rates compared between dates.

**Table 2:**
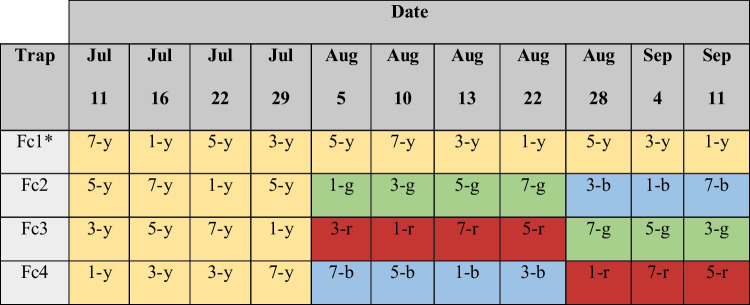
Trap sites (1, 3, 5, or 7) of the DIY Flebocollect light traps (Fc1 to Fc4) and colors of their diode light circuits (b, blue; g, green; r, red; or y, yellow). *Fc1 was used as an experimental control

### Processing and identification of sand flies

Sand flies were exposed to direct sunlight for 2 h and then separated from the contention nets with the help of a paintbrush and stored in ethanol 70% until identification. Each individual fly was identified to the species level following several phlebotomine sand flies taxonomic keys (Dantas-Torres et al. 2014; Tello-Fierro 2014).

### Data analysis

Rates of captured phlebotomines per Fc trap were calculated based on amounts of captured phlebotomines (classified by sex and species) compared to the average amount corresponding to the three control traps for each respective trapping night. Capture rates were then log-transformed for data analysis.

$$\mathrm{Fc}\;\mathrm{capture}\;\mathrm{rate}=\frac{\mathrm{Amounts}\;\mathrm{of}\;\mathrm{captured}\;\mathrm{phlebotomines}\;(\mathrm{Fc}\;\mathrm{traps})}{\mathrm{Average}\;\mathrm{captured}\;\mathrm{phlebotomines}\;(\mathrm{CDC}\;\mathrm{traps})}$$

$$\mathrm{Log}\;\mathrm{Fc}\;\mathrm{capture}\;\mathrm{rate}=\log_{10}\;(\mathrm{Fc}\;\mathrm{capture}\;\mathrm{rate}+1)$$

A Kruskal–Wallis test was used to compare logarithmic Fc1 capture rate totals between different capture nights. As no difference was observed between capture rates over time for Fc1, we concluded that there were no detrimental effects of time on the traps, and the matter is not further discussed (Kruskal–Wallis test, *p* = 0.2865; *n* = 11; df = 10).

As Fc1 was used as a control, the following analysis only takes into consideration traps Fc2, Fc3, and Fc4. The effectiveness of the Fc traps has been previously tested by our group (Gálvez et al. 2022). The normality and homogeneity of data were confirmed by the Lillieford and Bartlett tests respectively. A Kruskal–Wallis test was performed to examine the effect of the color of the light circuit (blue, green, red, and yellow) on Fc capture rate effectiveness. The impacts of the different light colors were compared using a Bonferroni post hoc test. Log-transformed rates of captured males and females, total *P. perniciosus* and *S. minuta*, and the sum of both species (total phlebotomines) were set as response variables. Significance was set at *p* ≤ 0.05. All tests were performed using RStudio Software, version 2021.9.0.351, employing the packages dplyr and ggplot2 for graph generation (RStudio Team 2020).

## Results

In total, 877 phlebotomine sand flies were captured, of which 865 individuals were identified to species level. Unidentified individuals were discarded from the data analysis (*n* = 12). Three species belonging to two different genera were found: 495 *Phlebotomus perniciosus* (353 males, 142 females), 359 *Sergentomyia minuta* (199 males, 160 females), and 11 *P. sergenti* Parrot, 1917 (10 males, 1 female). Mean captures per trap are provided below according to species, sex, and light color (Table 3).

**Table 3:** Mean captured phlebotomine sand flies classified by species (*Phlebotomus perniciosus*, *Sergentomyia minuta*, and total phlebotomines), sex (m, males; f, females, Tot, total), and light source color. A CDC incandescent halogen light trap was used as control. *Statistical differences

Color	Mean phlebotomines capture rate								
	*P. perniciosus*			*S. minuta*			Total phlebotomines		
	m	f	Tot	m	f	Tot	m	f	Tot
Control CDC	3.64	1.91	5.55	1.85	1.88	3.73	5.55	3.79	9.33
Blue Fc	4.29	2.57	6.86	2.86	1.14	4.00	7.57	3.71	11.29
Green Fc	1.86	1.86	3.71	4.00*	1.71	5.71	6.00	3.57	9.57
Red Fc	4.57	1.43	6.00	3.71*	3.71	7.43*	8.29	5.14	13.43
Yellow Fc	9.83	2.67	12.50	3.67	2.92	6.58	13.75	5.67	19.42

When we considered the light color used in the traps, no differences in total phlebotomines and *Phlebotomus perniciosus* captures were detected between the different colors. In the case of *Sergentomyia minuta*, differences were observed between colors in both total captures (Kruskal–Wallis test, *p* = 0,030; *n* = 132; df = 4) and males captured (Kruskal–Wallis test, *p* = 0.0128; *n* = 66; df = 4). Bonferroni’s post hoc test yielded the following results (Table 4).

**Table 4:** Bonferroni’s post hoc test for light color preference. Light source color was considered a categorical variable and logarithmic proportion capture rate a continuous variable. *Significant evidence (*p* value < 0.05)

Comparison	Males		Females		Total	
	*Z*	*p*	*Z*	*p*	*Z*	*p*
*Phlebotomus perniciosus*						
Control-blue	− 1.346	0.178	− 0.889	0.374	− 1.564	0.118
Control-green	− 0.255	0.799	1.193	0.233	0.688	0.492
Control-red	− 1.102	0.270	0.659	0.510	− 0.241	0.810
Control-yellow	− 1.025	0.305	0.367	0.713	− 0.413	0.679
*Sergentomyia minuta*						
Control-blue	− 0.807	0.420	1.183	0.237	0.186	0.853
Control-green	− 2.614	0.009*	1.356	0.175	− 1.018	0.309
Control-red	− 2.558	0.011*	− 1.037	0.300	− 2.743	0.006*
Control-yellow	− 0.882	0.378	0.565	0.572	− 0.318	0.750
*Total phlebotomines*						
Control-blue	− 0.744	0.457	0.138	0.890	− 0.404	0.686
Control-green	− 1.430	0.153	1.757	0.079	0.202	0.840
Control-red	− 1.637	0.102	0.027	0.979	− 1.220	0.222
Control-yellow	− 0.841	0.401	0.745	0.456	− 0.142	0.887

**Fig. 2:**
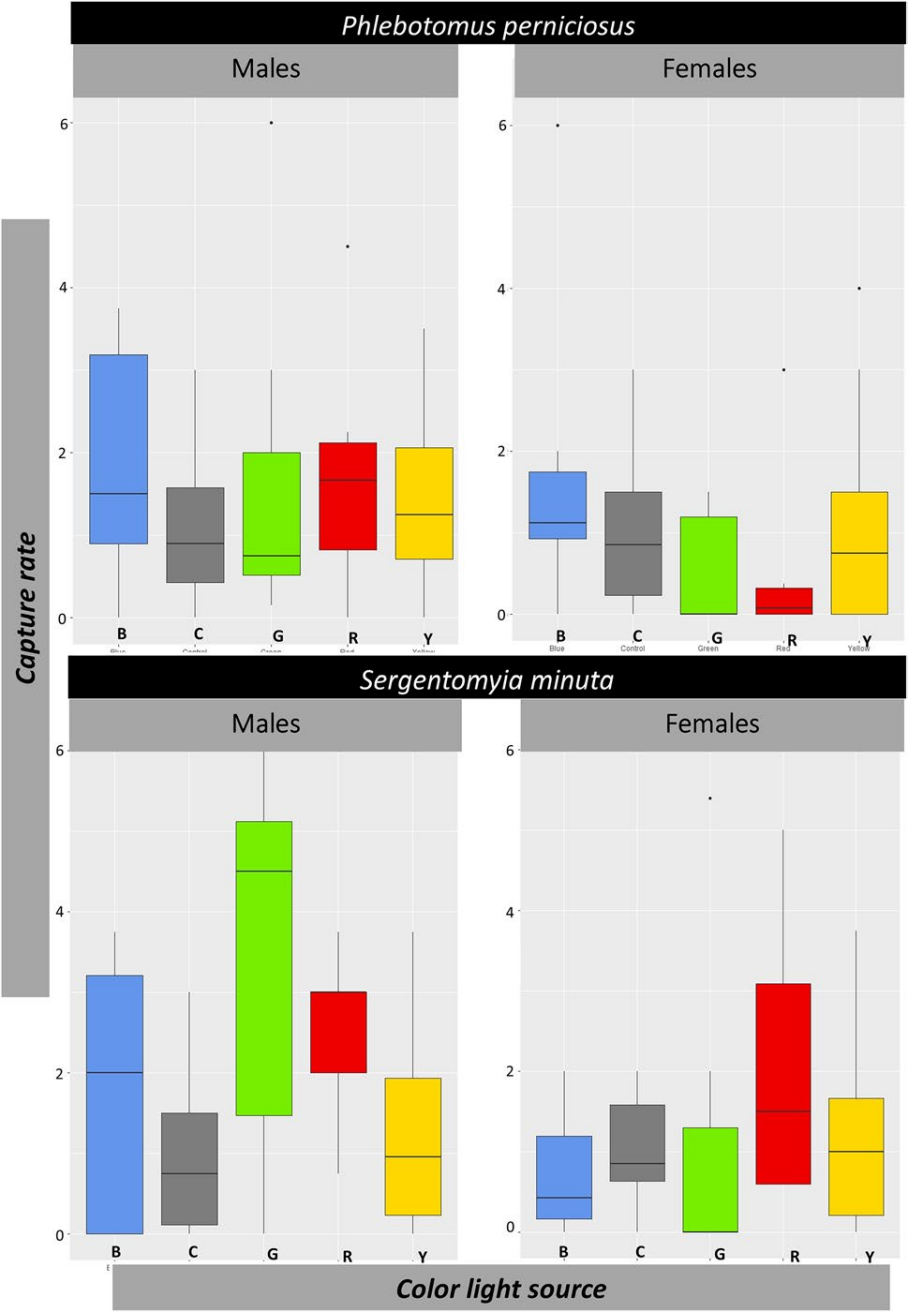
Boxplot showing the results of the descriptive analysis of phlebotomine sand fly preferences for different color lights (B, blue; C, control; G, green; R, red; Y, yellow respectively). *X*-axis, light color; *Y*-axis, capture rate. Median value, horizontal mid-line; minimum and maximum values, vertical line below and above the box, respectively; first and third percentiles, base and top of the box to the median line; outliers, atypical values represented as single points above the maximum line

These results indicate that *S. minuta* males are more likely to be lured by the green (Bonferroni test control-green; *Z* =  − 2.614, *p* value = 0.009) and red (Bonferroni test control-red; *Z* =  − 2.558, *p* value = 0.011) diode light sources of the Fc traps compared to those of the CDC control traps. Accordingly, mean capture rates (Table 3) were 2.16 (4.00/1.85) and 2.01 times greater (3.71/1.85) for the green and red light respectively compared to control CDC traps. Summarized capture rates of phlebotomine sand flies are shown Fig. 2.

The descriptive boxplot (Fig. 2) shows that, in the case of male *Sergentomyia minuta*, green and red light leads to a higher capture rate than recorded for control traps, as previously seen (Table 4). We should also highlight that, although without significance, traps emitting blue light returned higher capture rates than control traps in all cases except for *S. minuta* females. The data that support the findings of this study are available from the corresponding author upon reasonable request.

## Discussion

As seen in our previous studies, the most abundant phlebotomine species in the study area was *P. perniciosus* followed by *S. minuta* (Prieto et al. 2021). As *P. perniciosus* is the main vector of *L. infantum* in the Madrid region (Gálvez et al. 2010), it is essential to understand its biology and to optimize novel trapping methods to design future control programs for this vector.

In a recently published research, we observed no significant differences in capture rates between CDC and Fc light traps employing yellow diode light sources (Gálvez et al. 2022). Similar results were obtained comparing blue and green diode sources and incandescent lights in studies conducted in Brazil with modified light traps (da Silva et al. 2022). As such, all differences seen in the present study may be attributed to light colors. To our knowledge, this is the first time a color stimulus preference has been detected for any species of the genus *Sergentomyia*. We detected a preference of male *S. minuta* for green and red over the light emitted by control commercially available light traps. Traditionally, *S. minuta* has been considered to feed mainly on reptiles and has been held responsible for the transmission of *Leishmania tarentolae*, the causative agent of leishmaniasis in reptiles (González et al. 2020). Recent studies have examined the possibility that *S. minuta* could play a role in the transmission of *Leishmania* parasites to humans and other mammals (Maia and Depaquit 2016), as this sand fly has tested positive for *Leishmania major* (Campino et al. 2013) and *Leishmania infantum* (Pereira et al. 2017) (both cases reported in the Algarve region, Portugal) and has been identified as a more opportunistic feeder in blood screening studies in Madrid, Spain (González et al. 2020). However, more research is needed to describe *S. minuta* as a human parasite vector. Effectively, several criteria need to be fulfilled before a phlebotomine species is confirmed as a leishmaniasis vector (Ready 2012).

As described in Sect. 1.3, light color preference in phlebotomine sand flies is a controversial topic, as results to date have varied. In the literature, there are reports of a preference for both green (da Silva et al. 2020; Silva et al. 2015, 2016) and red (Hoel et al. 2007; Mann et al. 2009) light. The compound eye of *Lu. longipalpis* has two major light absorption peaks: ultraviolet (at 340 nm) and blue-green-yellow (at 520 nm for females and 546 nm for males) (Mellor et al. 1996). Accordingly, a green, blue, or even yellow light preference might be expected for other phlebotomine species. Further, the two light receptors could play a role in landmark navigation, and a UV-green contrast model has been proposed for insects (Möller 2002). We consider landmark navigation an essential feature of phlebotomines because sand flies, as nocturnal insects, need to identify their resting sites and to find a mate for reproduction and a feeding source at dusk or after dusk when light is scarce. UV and green-light detection could also play a role in the identification of plants as seen in other plant-feeding insects (Schnaitmann et al. 2020). As mentioned earlier, phlebotomines can feed from nectar, honeydew, or plant-sap (Abbasi et al. 2018). While no ultraviolet light source was employed here, it would be interesting to address its luring effectiveness in future work.

Although a preference for green light sources was expected, the observed preference also for red light was not. While Höel and colleagues in the case of *P. papatasi* (Hoel et al. 2007) and Mann and colleagues in work on *Lu. shannoni* (Mann et al. 2009) did detect a preference for red light, other studies on phlebotomines and other hematophagous dipterans have provided widely varying results (Table 1). In the biting midges *Culicoides*, a preference for blue, green, and ultraviolet light was reported (González et al. 2016) while red light sources were found to significantly underperform in comparison with other light sources (Hope et al. 2015). Based on *Drosophila* models, this preference could be explained by the types of photoreceptors present in the rhabdom of those flies, which are able to perceive mainly wavelengths of the yellow, blue, and UV spectrum (Schnaitmann et al. 2020). In contrast, as seen in early color preference studies, other hematophagous insects such as Simuliidae may be more attracted to dark colors such as blue, black, and even red (Bradbury & Bennett 1974). In the case of *Ae. aegypti*, a mosquito showing diurnal activity, chromacity and contrast are considered to play an important role in visual object attraction and human skin detection. Given that in mosquitoes rhabdomeres lack red-sensitive rhodopsin, red sources could be perceived as dark gray or black (Burkett & Butler 2005).

Background contrast could thus be useful for object detection, as is the case of human skin whose visualization is dominated by long wavelengths (van der Kooi et al. 2021). However, as phlebotomine sand flies are nocturnal insects, contrast between dark and light colors is considered less likely as an explanation for the red color preference shown by *S. minuta*, as is the case given for *Aedes* mosquitoes preferences (Burkett & Butler 2005). Thus, while light color seems to be an important visual stimulus, it is not the only one. Allan and colleagues determined that visual host identification by biting flies is the result of shape, size, color, contrast, light intensity, and texture (Allan et al. 1987). Despite this, light color has been the main variable examined in the phlebotomine research studies found in the literature (Table 1), although light intensity was also considered in several of these studies (Hoel et al. 2007; Mann et al. 2009; Silva et al. 2015). For Culicidae mosquitoes, it has even been suggested that light intensity could be a more important stimulus than color (Barr et al. 1963). To explore the effect of intensity, a neutral density filter was used to equalize the intensity of different wavelengths in a color preference study in culicine mosquitoes (Burkett & Butler 2005). Here, we did not assess the effect of light intensity, nor specified light wavelengths due to resource limitations, which should be better addressed in future studies. This should be taken into consideration in future studies. Moreover, besides visual signals, hematophagous insects combine olfactory, skin volatile, and thermal signals for host detection (Alexander 2000; van der Kooi et al. 2021). Other variables such as altitude (Chaniotis et al. 1971; Chaskopoulou 2018; Gaglio et al. 2014), environment (Chaskopoulou 2018; Gálvez et al. 2010), time of day (Chaskopoulou 2018; J. Lucientes et al. 2005), or moonlight (da Silva et al. 2020; Gaglio et al. 2014; Gebresilassie et al. 2015), can impact sand fly captures. As these conditions were similar for all traps and during each trapping night, they were not taken into consideration in the present study. Another variable that we would like to address is light reflection off the aluminum surface of our Fc traps. Indeed, Hoel et al. reported an attraction effect of light reflected off aluminum surfaces in phlebotomines (Hoel et al. 2007). A similar trend was described for *Ae. aegypti* and *Ae. albopictus* in that they preferred reflected over direct transmitted light. This was attributed to a greater need to detect resources as they are diurnal species (Burkett & Butler 2005). However, it does not explain the preference shown for reflected light by *P. papatasi* (Hoel et al. 2007) as being nocturnal insects. We should mention that the surface of the inside walls of the suction tunnel in the Fc light traps was composed of aluminum as the traps were made from recycled tetra bricks (Gálvez et al. 2022). This reflection could therefore be an attractant increasing their effectiveness over CDC traps, although this issue needs to be investigated further.

Flebocollect traps have been described as an alternative option to CDC traps for luring phlebotomine sand flies (Gálvez et al. 2022) and have also been suggested as a valid method of capturing culicine mosquitoes (López de Felipe et al. 2021). Besides being helpful, economic, and easy to manufacture, the Fc light trap is the outcome of the citizen science project “FleboCollect” proving the far-reaching potential of citizen science.

Autochthonous leishmaniasis caused by *Leishmania infantum* is the most important protozoan neglected tropical disease in southern European countries such as Spain (Hotez 2016). As the spread of leishmaniasis is expected to rise due to arising conflicts (Kamhawi 2017) and global warming (Gálvez et al. 2011), some action has to be taken. Being a zoonotic disease (Gálvez et al. 2018), leishmaniasis needs to be controlled under the concept of “One Health” (Gálvez et al. 2020). Understanding the biology and ecology of phlebotomine sand flies is essential for future monitoring and control programs for these vectors. Flebocollect light traps could be a great tool for future research projects designed to determine the sight characteristics and color preferences of hematophagous dipterans as a necessary step for optimizing control programs.

## Conclusions

A preference for green and red light was shown by male *Sergentomyia minuta* sand flies. To our knowledge, this is the first case of color preference reported in a species of the genus *Sergentomyia*. Despite being of great public health interest, the topic of color preference in hematophagous insects is a mostly untouched area of research. Flebocollect light traps emerged as useful tools. These DIY traps have several benefits: they are cheap, easy to manufacture, and have interchangeable and adjustable light sources. Although it is widely considered that dipterans can only perceive light in the yellow, ultraviolet, and blue spectra (short and medium wavelengths), our results suggest that phlebotomine sand flies could be able to detect and even be lured by red wavelengths of light.

## Further research

Light source brightness should be taken into consideration for future studies involving light color preferences. As such, filters could be employed to equalize different light intensities as proposed in the case of other hematophagous dipterans (Burkett & Butler 2005). The effect of direct and reflected light preferences should be evaluated in future studies. Anatomical and physiological studies of the anatomical structure of the phlebotomine sandfly’s compound eye should be undertaken to understand future and more complex responses to light sources, based on photoreceptor nature.

## Data Availability

The datasets generated during and/or analyzed during the current study are available from the corresponding author on reasonable request.
